# The complexity analysis of cerebral oxygen saturation during pneumoperitoneum and Trendelenburg position: a retrospective cohort study

**DOI:** 10.1007/s40520-022-02283-w

**Published:** 2022-11-02

**Authors:** Xiaoxiao Wang, Chang Liu, Kai Zhang, Yunliang Zhang, Yao Yu, Weidong Mi, Hao Li

**Affiliations:** 1 Research Center of Clinical Epidemiology, Peking University Third Hospital, 49 North Garden Road, Haidian District, Beijing, 100191 China; 2 Medical School of Chinese People’s Liberation, Army General Hospital (PLA), Beijing, 100853 China; 3 Department of Anesthesiology, The First Medical Center, Chinese PLA General Hospital, 28Th Fuxing Road, Haidian District, Beijing, 100853 China

**Keywords:** Cerebral oxygen saturation, Postoperative delirium, Brain complexity, Approximate entropy (ApEn), Sample entropy (SampEn)

## Abstract

**Background:**

The human brain is a highly complex and nonlinear system, nonlinear complexity measures such as approximate entropy (ApEn) and sample entropy (SampEn) can better reveal characteristics of brain dynamics. However, no studies report complexity of perioperative physiological signals to reveal how brain complexity associates with age, varies along with the development of surgery and postoperative neurological complications.

**Aim:**

This study examined the complexity of intraoperative regional cerebral oxygen saturation (rSO_2_), aiming to reveal brain dynamics during surgery.

**Methods:**

This retrospective cohort study enrolled patients who scheduled for robot-assisted urological surgery. Intraoperative rSO_2_ was continuously monitored throughout the surgery. Postoperative delirium (POD) was diagnosed by the Confusion Assessment Method. ApEn and SampEn were used to characterize the complexity of rSO_2_. Pearson correlation coefficients were used to measure the correlation between complexity of rSO_2_ and age. The association between complexity of rSO_2_ and POD was examined using *T* tests.

**Results:**

A total of 68 patients (mean [SD] age, 63.0 (12.0) years; 47 (69.1%) males) were include in this analysis. There was a significant reverse relationship between the complexity of rSO_2_ and age (The correlation coefficients range between − 0.32 and − 0.28, all *p* < 0.05). Patients  ≥ 75 years showed significantly lower complexity of rSO_2_ than the other two groups. Older age remained an independent factor influencing complexity of rSO_2_ after adjusting for a number of covariates. Six patients (8.8%) developed POD, and POD patients had lower complexity of rSO_2_ compared with non-POD patients.

**Conclusions:**

The complexity of rSO_2_ may serve as a new candidate marker of aging and POD prediction.

**Supplementary Information:**

The online version contains supplementary material available at 10.1007/s40520-022-02283-w.

## Introduction

Cerebral autoregulation is an important defense mechanism of the brain against hypo- and hyper-perfusion, ensuring the stability of cerebral blood flow over a specific range of fluctuations in systemic hemodynamics [1]. However, impaired cerebral autoregulation might be observed in some surgeries with special positions. The steep Trendelenburg position which is mandatory for visual inspection in the robot-assisted surgery, may have effects on cerebral and systemic hemodynamic fluctuation, more prone to suffer hypoxia and ischemia. Elevated venous return and increased intracranial pressure can be investigated in the reverse Trendelenburg position, triggering overperfusion, dysregulation and even complications, such as stroke, cerebral hemorrhage and postoperative delirium (POD). Meanwhile, CO_2_ pneumoperitoneum during the robot-assisted surgery has effect on cerebral vasculature and increased pneumoperitoneum pressure, resulting in cerebral autoregulation deterioration. Therefore, patients under the steep Trendelenburg position with pneumoperitoneum for several hours own less effective cerebral autoregulation and are more vulnerable to neurological complications.

Recent years, the application of non-invasive near infrared spectroscopy (NIRS) offers a new insight for monitoring cerebral oxygen saturation in order to reflect cerebral perfusion and oxygen metabolic status in a continuous and real-time method [2, 3]. However, conventional liner measurements and analysis mostly focus on the mean and lowest regional cerebral oxygen saturation (rSO_2_) value during the intraoperative period, unable to reveal the underlying complexity of the brain dynamics and thus losing the nonlinear features in complex systems. The human brain is a complex non-linear system with high sensitivity to small fluctuations. Therefore, nonlinear approaches to explore the complex system of brain have been considered by researchers for the analysis of complex brain states [4]. Several studies have found that nonlinear analysis can be used to facet physiological signals, and there are studies suggesting that physiological signal complexity decreases with age and correlates with disease and anesthesia state [5]. In trauma patients, Riordan et al. [6] have demonstrated reduced heart rate complexity was associated with poor outcomes. Lu et al. [7] found that complexity index of intracranial pressure was an independent predictor of unfavorable death among patients after traumatic brain injury. However, until now, no nonlinear analysis has been applied to perioperative physiological signals to reveal how the cerebral oxygen saturation complexity associates with age, varies along with the development of surgery and POD outcome, and characterizes the degree of disorder and chaos of non-stationary signals.

Therefore, our study intended to analyze the relationship between cerebral oxygen saturation complexity with different ages, synchronization of surgery and the unfavorable POD outcome, thus promoting the use of nonlinear method, in the analysis of physiological signals, especially during the intraoperative period [8]. Approximate entropy (ApEn) and sample entropy (SampEn) were introduced to our study, which were both indicators to quantify the complexity of functional brain activity [9]. We further hypothesized that the application of complexity of brain NIRS signals may provide strategies for preventing cerebral events in clinical practice.

## Materials and methods

### Study population

Patients who scheduled for robot-assisted urological surgery under general anesthesia in the First Medical Centre of the PLA General Hospital between April and October 2019 were enrolled. The types of surgery included robotically assisted prostatectomy and cystectomy which both require the establishment of artificial pneumoperitoneum and steep Trendelenburg position. Patients older than 18 years were included in our study. The exclusion criteria included patients who met the American Society of Anesthesiologists (ASA) grade IV or above, with history of vital organ disease (brain trauma, cerebrovascular disease, mental disorders, endocrine or metabolic disease, severe heart or lung disease, liver or kidney insufficiency, or severe malnutrition), psychotropic drug usage over the long term; and a preoperative mini-mental state examination (MMSE) score less than 24 points. Patients with incomplete data were excluded as well. The study was a secondary analysis of previously collected data, approved by Research Ethics Committee of PLAGH (No. S2019-120-02), and also consistent with the declaration of Helsinki. Informed consent forms were obtained from each patient or a family member.

### Intraoperative management

Standard monitoring was performed upon admission, including electrocardiogram (ECG), noninvasive blood pressure (NIBP), pulse oximetry, and bispectral index (BIS). Before induction of anesthesia, bilateral sensors are placed on each side of forehead above the eyebrows to record cerebral oxygen saturation (rSO_2_) by INVOS (5100c, Covidien, USA). RSO_2_ was continuously monitored every 6 s in both sides throughout the whole anesthesia period. Peripheral venous access and peripheral arterial cannulation were also required prior to induction of anesthesia. After opening the peripheral veins, midazolam (0.06 mg/kg) was injected for sedation. Drugs for induction of anesthesia: propofol (2 mg/kg), sufentanil (0.3 μg/kg) and rocuronium (0.6 mg/kg). A lung-protective ventilation strategy was used after tracheal intubation, with a tidal volume of 6–8 ml/kg, a respiratory rate of 12–15/min, an inspiratory to expiratory ratio of 1:2, and an oxygen content of 60% of the inhaled gas. The fresh gas flow rate was 2 L/min to maintain PaCO_2_ between 35 and 45 cmH_2_O. Anesthesia was maintained with intravenous pumping of propofol and remifentanil supplemented with sevoflurane inhalation. The minimum alveolar concentration (MAC) value of sevoflurane was maintained < 1 and propofol (4–8 mg/kg/h) and remifentanil (0.1–0.2 μg/kg/min) were continuously pumped to maintain BIS between 40 and 60, maintain blood pressure and heart rate within ± 20% of basal, and use vasoactive drugs as appropriate. Intraoperative hemostasis was completed with the placement of drainage when sevoflurane was turned off to take full intravenous anesthesia maintenance, supplemented with sufentanil (0.1–0.2 μg/kg) as needed, and propofol and remifentanil pumping were stopped before the end of abdominal closure. Continuous arterial pressure, urine output, partial pressure of end-of-breath carbon dioxide (PETCO_2_) as well as rSO_2_ were monitored and recorded. The rSO_2_ in the left side was represented as channel 1 (ch1) and the right one as channel 2 (ch2). All patients were transferred to the anesthesia care unit (PACU) for postoperative monitoring. As regards intraoperative settings, after anesthesia induction is complete, a pneumoperitoneal needle is inserted and CO_2_ is charged at a gas flow rate of 14 L/min to bring the pneumoperitoneal pressure to 12 mmHg. After the pneumoperitoneum is successfully established, the patient is adjusted to a steep Trendelenburg position (30° head‐down tilt). Due to the requirement for full exposure of the surgical field, surgical pneumoperitoneum pressure is generally maintained at 12–14 mmHg (1 mmHg = 0.133 kPa), CO_2_ flow rate is maintained at 3–5 L/min, until the end of the procedure before closing the pneumoperitoneum and adjusting the patient back to the supine position.

### POD assessment

Clinical data throughout the study period were collected. In the first five postoperative days, an anesthesiologist or anesthesia nurse who was not involved in the anesthesia procedure and was trained with the diagnostic criteria of delirium evaluated the patients for POD using the Confusion Assessment Method (CAM) [10].

### Intraoperative rSO_2_

In healthier individuals, the human body strived to create balance between brain tissue oxygen consumption and supply to support fully functioning brain systems and optimize health. More engagement of the regulatory components, and more mobilized metabolic units were expected in healthier individuals in response to stressors, indicating more information processing. As entropy (e.g., ApEn or SampEn) is a measure of information content in complex physiologic time series, healthier individuals presented higher entropy values of physiological signals (e.g., intraoperative rSO_2_). ApEn and SampEn are nonlinear measures used for assessing the regularity and unpredictability of physiological time-series signals, diagnosing diseased states [11]. ApEn and SampEn values are non-negative, with lower values corresponding to greater regularity indicating greater component autonomy and isolation (manifested as crucial biologic messages slow to transmit and receive or unable to arrive). They are applicable to relatively short (greater than 100 points) and noisy time-series data. ApEn and SampEn have better performance than moment statistics (such as mean and variance) and rank-order statistics (such as median and interquartile range) in discriminating series for which clear feature recognition is difficult. ApEn and SampEn reflect the likelihood that runs of patterns that are close (within *r*) form contiguous observations remain close (within the same tolerance *r*) on subsequent incremental comparisons (pattern length *m* + 1). The difference between two statistics is that SampEn does not include self-similar patterns as ApEn does. The parameters, *m* and *r* were used for the calculations: *m* is the length of compared runs, and *r* is effectively a filter to define the similar pattern. It is recommended to set *m* to 2 and *r* to 0.2 times the SD of the original time series [12]. In the present study, we applied ApEn and SampEn to intraoperative rSO_2_ signals, with *m* = 2 and *r* equal to 0.2 times the SD of the original time series. The rSO_2_ time-series data during each operation (of length *n*) were segmented into five subset time-series data of length *n*/5. The calculations were conducted to each time-series data set.

### Sample size

A sample size of 61 achieves 80% power to detect a difference of − 0.35 between the null hypothesis correlation of 0.0 and the alternative hypothesis correlation of 0.35 using a two-sided hypothesis test with a significance level of 0.05. Given an anticipated dropout rate of 5%, total sample size required is 65.

### Statistical analysis

IBM SPSS Statistics for Windows, version 26.0 (IBM Corp., Armonk, N.Y., USA) and GraphPad Prism version 8.0.0 for Windows were used for statistical analyses. Categorical variables are presented as *n* (%). Continuous data with normal distribution are expressed as mean (SD), non-normal variables were reported as median and quartiles. Pearson correlation coefficients were used to measure the correlation between the ApEn or SampEn values of intraoperative rSO_2_ time-series data and age. Multiple linear regression was performed to estimate the association while adjusting for potentially confounding variables. A P value less than 0.05 indicates statistical significance.

## Results

### Subject characteristics

A total of 80 patients who underwent robot-assisted urological surgery at our hospital between April and October 2019 met the inclusion criteria. Twelve patients were excluded: reasons were incomplete intraoperative data. The remaining 68 patients were included in this analysis, six patients (8.8%) developed postoperative delirium. Table 1 shows the baseline characteristics of patients. Approximately 69% (47 of 68) patients were males. The patients’ mean age was 63.0 years (SD = 12.0), and the average BMI was 25.1 kg/m^2^ (± 3.0). Approximately 43% of patients were diagnosed with hypertension, 14.7% had diabetes mellitus, 64.7% of patients had hyperlipidemia, and 20.6% of patients had atrial fibrillation history. Additionally, 69% underwent prostatectomy, and 31% underwent cystectomy. The head-down position was kept for 150 (120, 190) minutes during operation.

**Table 1:** Baseline characteristics of patients (*n* = 68)

Characteristics	Description
Male	47 (69.1)
Age (years)	63.0 ± 12.0
BMI (kg/m^2^)	25.1 ± 3.0
Hypertension	29 (42.6)
Diabetes	10 (14.7)
Hyperlipidemia	44 (64.7)
Atrial fibrillation	14 (20.6)
Smoking	38 (55.9)
Head-down time (min)	150 (120, 190)
Urine volume (mL)	450 (200, 525)
Hemorrhage (mL)	100 (50, 200)
Fluid volume (mL)	2200 (1600, 3100)
Postoperative delirium	6 (8.8)

### The effect of aging on the complexity of rSO_2_

**Fig. 1:**
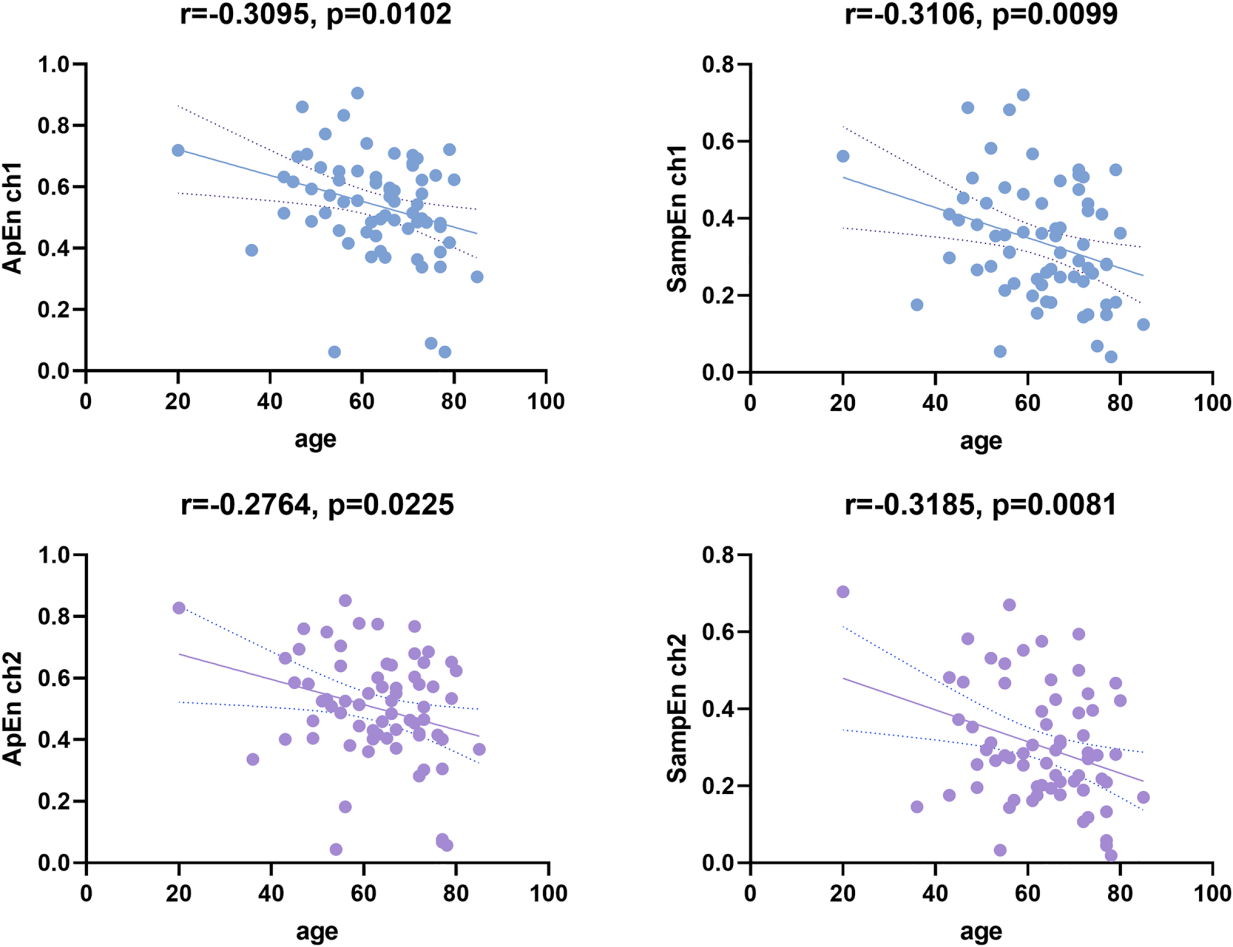
Correlation analysis between complexity analysis of intraoperative rSO_2_ and age. *rSO*_*2*_ regional cerebral oxygen saturation, *ApEn* approximate entropy, *SampEn* sample entropy, *ch1* the rSO_2_ of the left side, *ch2* the rSO_2_ of the right side

The mean approximate entropy was 0.540 ± 0.164 and 0.502 ± 0.178 for ch1 and ch2, and the sample entropy was 0.338 ± 0.152 and 0.303 ± 0.156, respectively. The relationship between entropy measures and age was tested (Fig. 1). The graph showed there was a significant linear relationship between the complexity of rSO_2_ and age (all *p* < 0.05). It showed that increasing age is indicative of a decreased complexity of cerebral oxygen saturation. Age remained independent factors influencing complexity of rSO_2_ (all *p* < 0.01) after adjusting for potential confounding factors, including sex, BMI and history of chronic illness (Supplementary Table 1).

According to age, total 68 patients were also grouped, 34 (50.0%), 23 (33.8%), and 11 (16.2%) patients were in the three age groups: middle-age group, advanced-age group and super advanced-age group (≤ 65, 65–74,  ≥ 75 years). As shown in Supplementary Fig. 1 and Table 2, there was no significant difference between the middle-age group and the advanced-age group measured by both approximate and sample entropy (ch1: *p* = 0.493 in approximate entropy; *p* = 0.408 in sample entropy; ch2: *p* = 0.725 in approximate entropy; *p* = 0.377 in sample entropy). Statistically, the super advanced-age group (≥ 75) showed significantly lower approximate entropy values in comparison with other two groups (ch1: *p* = 0.003 compared with middle-age group and *p* = 0.021 with advanced-age group; ch2: *p* = 0.008 compared with middle-age group and *p* = 0.022 with advanced-age group, respectively), indicative of a more regular pattern of cerebral oxygen saturation.

### The complexity of rSO_2_ in patients with or without POD

Supplementary Fig. 2 displays the complexity of rSO_2_ in the POD and non-POD groups. Although there was no statistical difference between groups, POD patients had lower entropy values (ApEn and SampEn) compared with non-POD patients. The mean approximate entropy and sample entropy were 0.43 ± 0.22 and 0.24 ± 0.13 for ch1 in those who developed POD, whereas the corresponding measures were 0.55 ± 0.16 and 0.35 ± 0.15 in those without POD (*p* = 0.081 and 0.086). The mean approximate entropy and sample entropy were 0.36 ± 0.27 and 0.22 ± 0.20 for ch2 in POD patients, while the entropy statistics of ch2 were 0.52 ± 0.16 and 0.31 ± 0.15 in non-POD patients (*p* = 0.232 and 0.177).

### The complexity of rSO_2_ over time

**Fig. 2:**
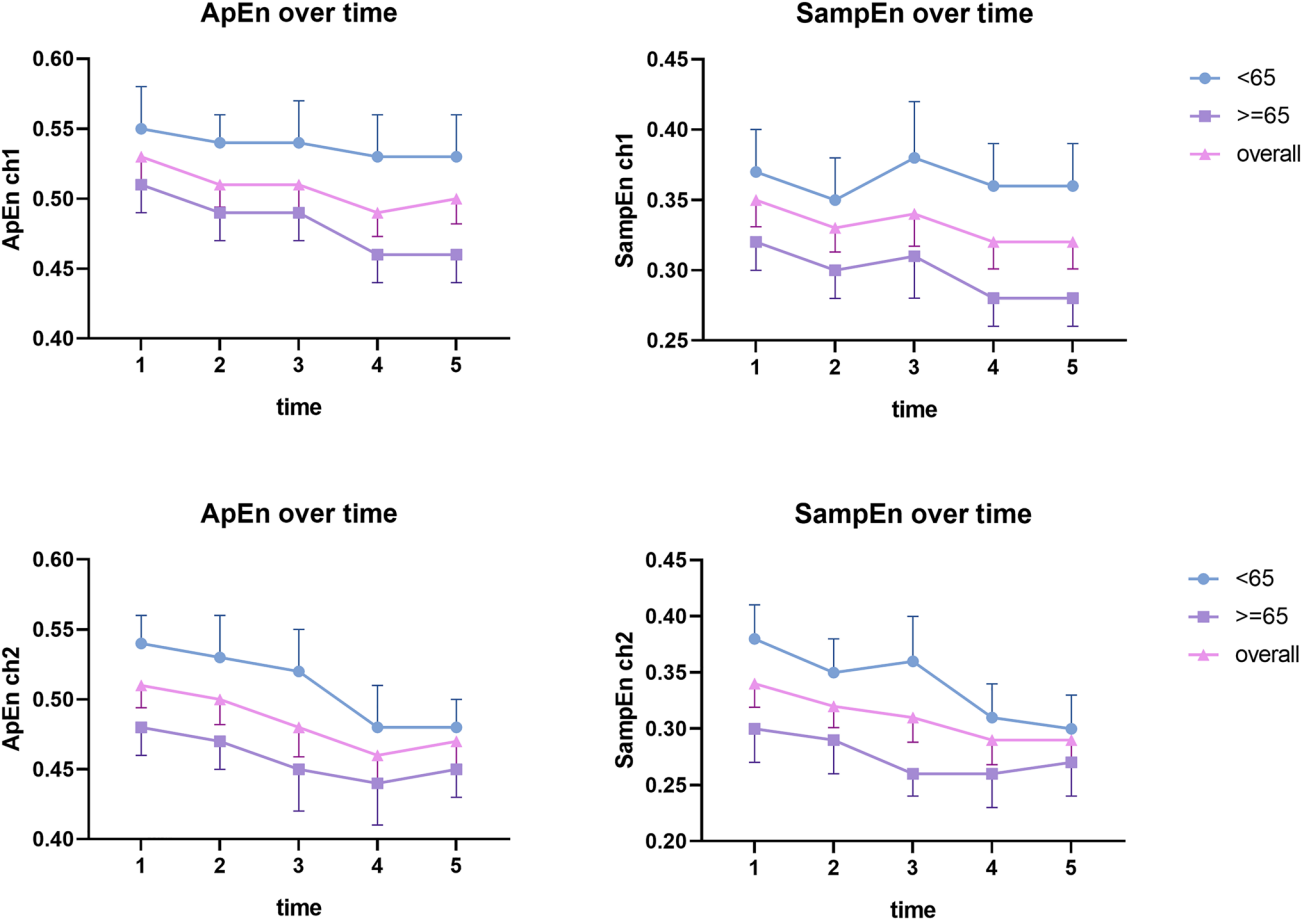
Complexity analysis of intraoperative rSO_2_ along with the development of surgery. *rSO*_*2*_ regional cerebral oxygen saturation, *ApEn* approximate entropy; *SampEn* sample entropy, *ch1* the rSO_2_ of the left side, *ch2* the rSO_2_ of the right side

**Fig. 3:**
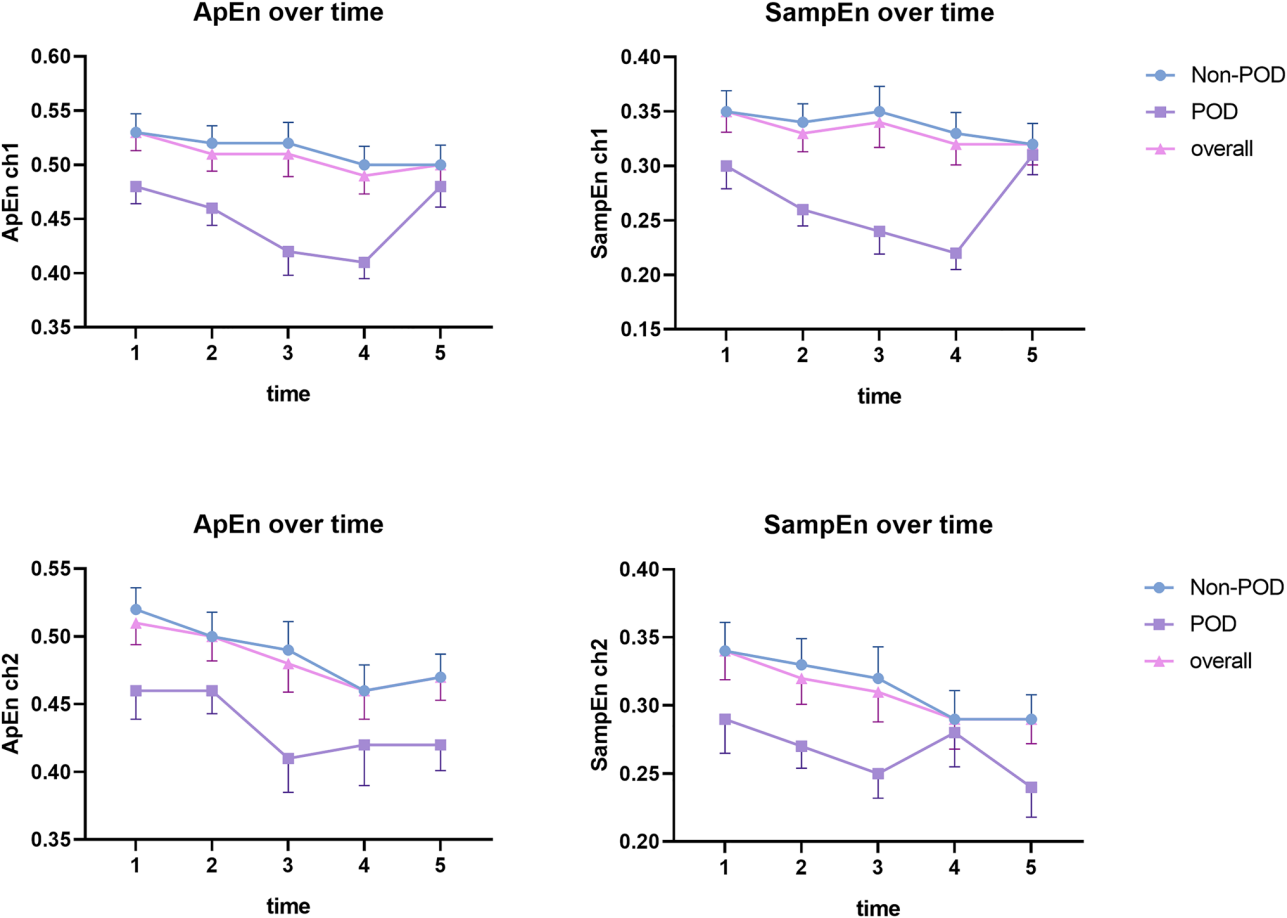
Complexity analysis of intraoperative rSO_2_ along with the development of surgery in POD and non-POD patients. *POD* postoperative delirium, *rSO*_*2*_ regional cerebral oxygen saturation, *ApEn* approximate entropy, *SampEn* sample entropy, *ch1* the rSO_2_ of the left side, *ch2* the rSO_2_ of the right side

Figures 2 and 3 display the complexity of rSO_2_ over time grouping in two ways but the complexity of rSO_2_ decreased with the progressing of the operation in both groups and both channels. The surgical process was divided into five phases and it can be observed that the mean approximate entropy and sample entropy value began to show a downward trend with the surgery processing in middle-age group (< 65) and elderly group (≥ 65). In addition, POD patients showed decreased complexity of rSO_2_ throughout the entire operation (Supplementary Fig. 3).

## Discussion

In this observational study, we exploited a novel non-liner measure to explore the relationship between intraoperative cerebral oxygen saturation and age, POD. The complexity of cerebral oxygen saturation reducing with age was observed. The finding suggests greater component autonomy and isolation that occurs with aging. The complexity of physiological signals may serve as a promising lead toward the measure of cerebral function of developmental age. The complexity of rSO_2_ was also reduced in POD patients, which provides a candidate indicator for POD early prediction.

It has been indicated that the human brain is a highly complex and nonlinear system, analysis of the brain using only traditional methods such as moment statistics and rank-order statistics cannot reveal all the characteristics of brain dynamics [13]. Thus, rSO_2_ signals can be better characterized by nonlinear dynamic measures, such as approximate entropy and sample entropy, which have the ability to monitor cerebrovascular transient reactivity to the environment changes. Decreased complexity can be investigated in several diseases and ageing and considered as a signal of failure in regulation.

In the present study, the results indicated older age is indicative of a decreased complexity of rSO_2_ signal. This is interpreted as a decrease in complexity that occurs with aging. Similar results have been shown in previous studies. Angelini et al. [14] reported that complexity of heart rate for the young population was significantly higher than that of the older population. Bhogal et al. [15] observed that a significant reduction in complexity of pulse oxygen saturation with age. The lower approximate entropy and sample entropy does not only suggest reduced complexity, but also might suggest increased system isolation, which may reflect partial “uncoupling” of the control system.

According to Pincus [16], healthier or stronger individuals have good line of communication, which is characterized by high complexity of physiological signals. However, crucial biologic messages in aging, frail, and diseased individuals are either slow to transmit and receive or unable to arrive, which is characterized by a more regular pattern of physiological signals. Our result may be indicative of the partial uncoupling of the systems involved in the cerebral oxygen metabolism through aging. The complexity of rSO_2_ decreased with the progress of surgery, suggesting that particular attention should be paid to anesthesia management in the middle and late period of surgery.

POD is a common postoperative complication, associated with increased mortality. Appropriate monitoring and early recognition of POD are essential, and the association between cerebral oxygen desaturation and POD have been studied widely. Nevertheless, the findings were not always conclusive. According to the above-mentioned inconsistent results about rSO_2_ and POD risk, we still do not know whether rSO_2_ contributes to POD onset. Existing literature uses absolute and relative declines in rSO_2_ to establish a link between rSO_2_ and POD. However, these measurements discard a lot of information and therefore do not characterize NIRS-based rSO_2_ well, better measures are needed to characterize rSO_2_. Non-linear dynamical analysis is a powerful approach for understanding physiological signals. Approximate entropy and sample entropy are applicable to relatively short and noisy time-series data, and have better performance than traditional methods for which clear feature recognition is difficult.

In this study, non-linear analysis was used to evaluate intraoperative rSO_2_ to provide more comprehensive understanding for cerebral status. We observed that POD patients had lower entropy values compared with non-POD patients. The result did not meet the predetermined level of significance owing to the small sample size in the POD group, but was close. The complexity of rSO_2_ can serve as an alternative method to assess the reactivity of brain and show promise as a candidate marker for POD prediction. Further study is needed to look at linear and non-linear indices of rSO_2_ in the prediction of POD.

A potential limitation of the study is that the retrospective nature and was conducted in a single center in Beijing, China. The small sample size in the POD group limited us to draw a conclusion about the association between complexity of rSO_2_ and POD.

In conclusion, the present study has established a decrease in complexity of rSO_2_ that occurs with aging. In addition, reduction in complexity of rSO_2_ may serve as a new candidate marker of aging, and POD.

## Supplementary Information

Below is the link to the electronic supplementary material.

Supplementary file1 (DOCX 17 KB)

Supplementary file2 The complexity analysis of intraoperative rSO_2_ in different age groups (34, 23, and 11 patients were in the three age groups, respectively). rSO_2_, regional cerebral oxygen saturation; ApEn, approximate entropy; SampEn, sample entropy; ch1, the rSO_2_ of the left side; ch2, the rSO_2_ of the right side (TIF 477 KB)

Supplementary file3 The complexity analysis of intraoperative rSO_2_ in POD and non-POD patients. POD, postoperative delirium; rSO_2_, regional cerebral oxygen saturation; ApEn, approximate entropy; SampEn, sample entropy; ch1, the rSO_2_ of the left side; ch2, the rSO_2_ of the right side (TIF 146 KB)

Supplementary file4(PNG 375 KB)

Supplementary file5 (PNG 372 KB)
