# Supplementary material for: The complexity analysis of cerebral oxygen saturation during pneumoperitoneum and Trendelenburg position: a retrospective cohort study
Source: Aging Clin Exp Res. 2022 Nov 2;35(1):177–84. doi: 10.1007/s40520-022-02283-w (PMC9816202; doi:10.1007/s40520-022-02283-w)
Supplement: Supplementary file 1 — Supplementary file1 (DOCX 17 KB) [file 40520_2022_2283_MOESM1_ESM.docx]

Supplementary Table 1. The effect of aging on the complexity of rSO_2_

| dependent | independent | B | SE | t | p |
| --- | --- | --- | --- | --- | --- |
| ApEn ch1 | constant | 0.513 | 0.209 | 2.457 | 0.017 |
|  | Age | -0.006 | 0.002 | -3.144 | 0.003 |
|  | Hypertension | 0.016 | 0.038 | 0.409 | 0.684 |
|  | Diabetes | -0.061 | 0.055 | -1.116 | 0.269 |
|  | Smoking | -0.017 | 0.046 | -0.362 | 0.718 |
|  | Hyperlipidemia | -0.039 | 0.046 | -0.857 | 0.395 |
|  | Atrial fibrillation | 0.050 | 0.046 | 1.085 | 0.283 |
|  | BMI | 0.021 | 0.007 | 3.047 | 0.003 |
|  | Female | -0.096 | 0.050 | -1.941 | 0.057 |
| SampEn ch1 | constant | 0.421 | 0.200 | 2.107 | 0.039 |
|  | Age | -0.005 | 0.002 | -3.022 | 0.004 |
|  | Hypertension | 0.009 | 0.037 | 0.240 | 0.811 |
|  | Diabetes | -0.049 | 0.053 | -0.934 | 0.354 |
|  | Smoking | -0.013 | 0.044 | -0.283 | 0.778 |
|  | Hyperlipidemia | -0.019 | 0.044 | -0.439 | 0.662 |
|  | Atrial fibrillation | 0.028 | 0.044 | 0.642 | 0.523 |
|  | BMI | 0.015 | 0.007 | 2.252 | 0.028 |
|  | Female | -0.092 | 0.047 | -1.930 | 0.058 |
| ApEn ch2 | constant | 0.694 | 0.242 | 2.868 | 0.006 |
|  | Age | -0.006 | 0.002 | -2.945 | 0.005 |
|  | Hypertension | 0.005 | 0.044 | 0.108 | 0.914 |
|  | Diabetes | 0.020 | 0.064 | 0.314 | 0.755 |
|  | Smoking | -0.027 | 0.054 | -0.497 | 0.621 |
|  | Hyperlipidemia | 0.025 | 0.053 | 0.474 | 0.637 |
|  | Atrial fibrillation | 0.057 | 0.054 | 1.068 | 0.290 |
|  | BMI | 0.011 | 0.008 | 1.427 | 0.159 |
|  | Female | -0.091 | 0.057 | -1.587 | 0.118 |
| SampEn ch2 | constant | 0.576 | 0.209 | 2.755 | 0.008 |
|  | Age | -0.006 | 0.002 | -3.339 | 0.001 |
|  | Hypertension | -0.007 | 0.038 | -0.180 | 0.858 |
|  | Diabetes | 0.036 | 0.055 | 0.656 | 0.514 |
|  | Smoking | -0.016 | 0.047 | -0.342 | 0.733 |
|  | Hyperlipidemia | 0.003 | 0.046 | 0.058 | 0.954 |
|  | Atrial fibrillation | 0.056 | 0.046 | 1.210 | 0.231 |
|  | BMI | 0.008 | 0.007 | 1.167 | 0.248 |
|  | Female | -0.087 | 0.050 | -1.743 | 0.086 |

rSO_2_, regional cerebral oxygen saturation; ApEn, approximate entropy; SampEn, sample entropy; ch1, the rSO_2_ of the left side; ch2, the rSO_2_ of the right side.

Supplementary Table 2. The complexity of rSO_2_ in different age groups

|  | (I) | (J) | Difference (I-J) | p | 95% Confidence Interval | |
| --- | --- | --- | --- | --- | --- | --- |
| Ap ch1 | <65 | 65-74 | 0.02897 | 0.493 | -0.055 | 0.113 |
|  | <65 | ≥75 | .16456* | 0.003 | 0.0566 | 0.2725 |
|  | 65-74 | ≥75 | .13559* | 0.021 | 0.0215 | 0.2496 |
| Samp ch1 | <65 | 65-74 | 0.03301 | 0.408 | -0.0462 | 0.1122 |
|  | <65 | ≥75 | .13450* | 0.01 | 0.0327 | 0.2363 |
|  | 65-74 | ≥75 | 0.10149 | 0.064 | -0.0061 | 0.2091 |
| Ap ch2 | <65 | 65-74 | 0.01631 | 0.725 | -0.0759 | 0.1086 |
|  | <65 | ≥75 | .16363* | 0.008 | 0.0451 | 0.2822 |
|  | 65-74 | ≥75 | .14732* | 0.022 | 0.0221 | 0.2726 |
| Samp ch2 | <65 | 65-74 | 0.03641 | 0.377 | -0.0453 | 0.1181 |
|  | <65 | ≥75 | .12615* | 0.019 | 0.0212 | 0.2311 |
|  | 65-74 | ≥75 | 0.08974 | 0.111 | -0.0212 | 0.2006 |

rSO_2_, regional cerebral oxygen saturation; ApEn, approximate entropy; SampEn, sample entropy; ch1, the rSO_2_ of the left side; ch2, the rSO_2_ of the right side.
