# Supplementary figures and images for: The complexity analysis of cerebral oxygen saturation during pneumoperitoneum and Trendelenburg position: a retrospective cohort study
Source: Aging Clin Exp Res. 2022 Nov 2;35(1):177–84. doi: 10.1007/s40520-022-02283-w (PMC9816202; doi:10.1007/s40520-022-02283-w)

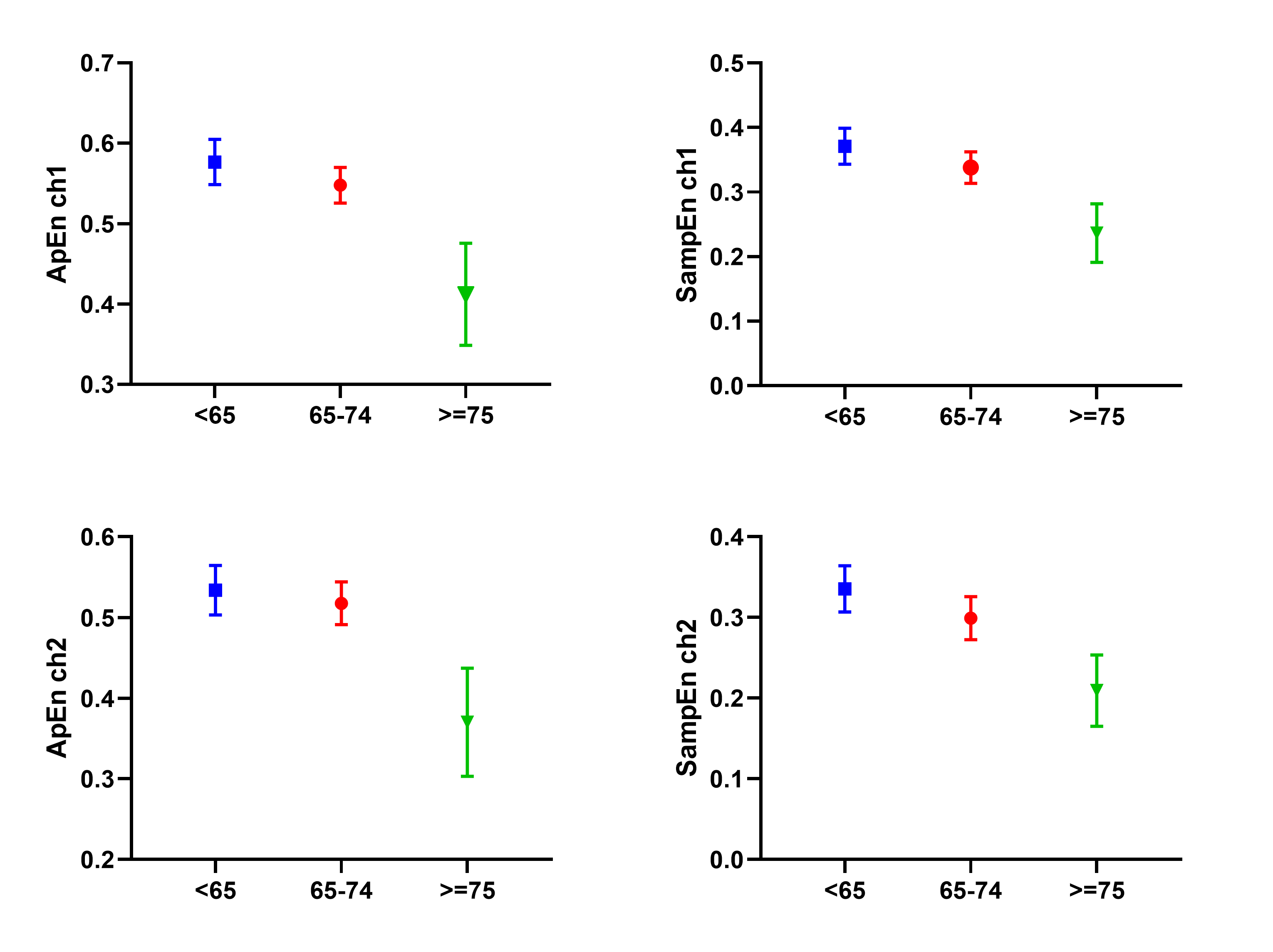

Supplement: Supplementary file 2 — Supplementary file2 The complexity analysis of intraoperative rSO2 in different age groups (34, 23, and 11 patients were in the three age groups, respectively). rSO2, regional cerebral oxygen saturation; ApEn, approximate entropy; SampEn, sample entropy; ch1, the rSO2 of the left side; ch2, the rSO2 of the right side (TIF 477 KB) [file 40520_2022_2283_MOESM2_ESM.tif]

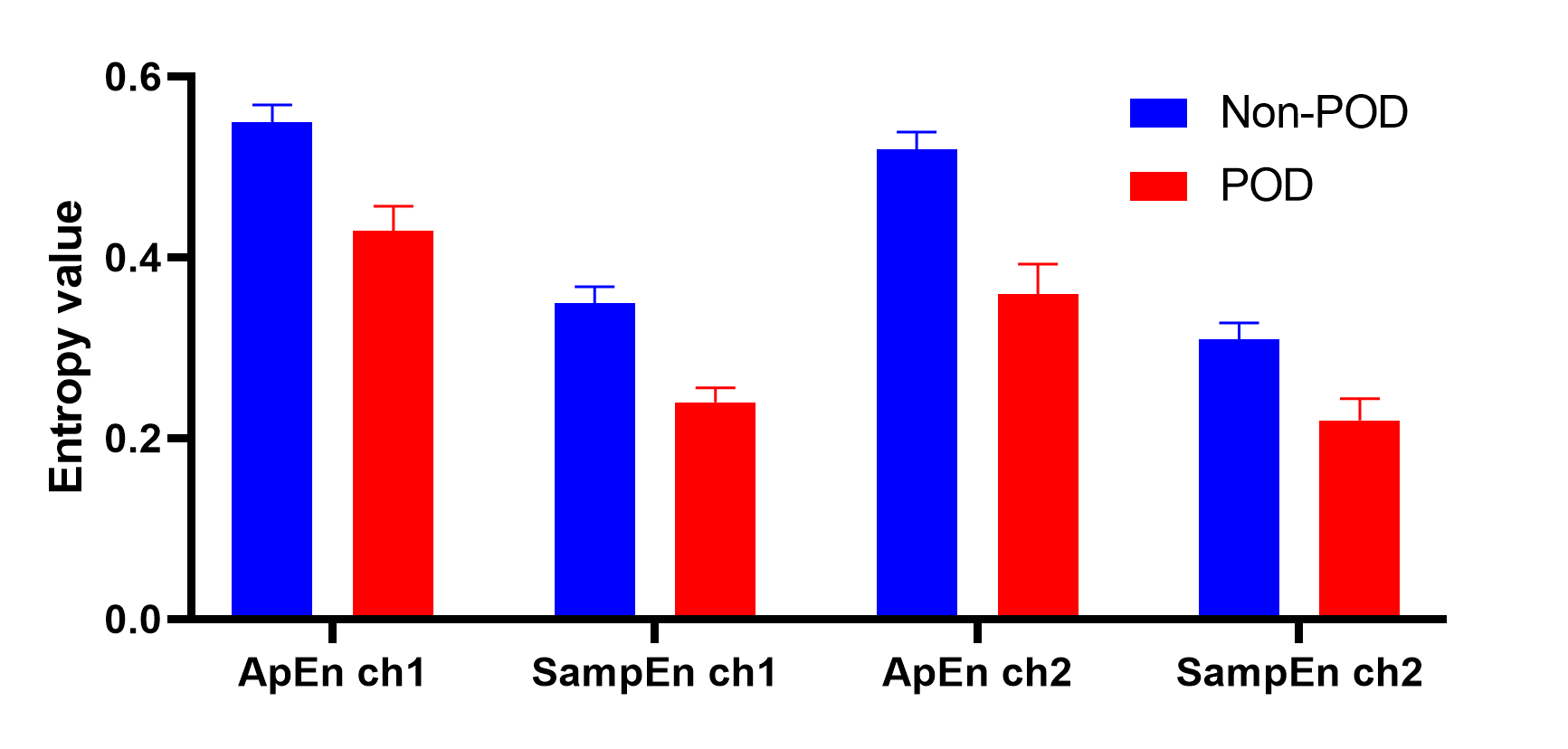

Supplement: Supplementary file 3 — Supplementary file3 The complexity analysis of intraoperative rSO2 in POD and non-POD patients. POD, postoperative delirium; rSO2, regional cerebral oxygen saturation; ApEn, approximate entropy; SampEn, sample entropy; ch1, the rSO2 of the left side; ch2, the rSO2 of the right side (TIF 146 KB) [file 40520_2022_2283_MOESM3_ESM.tif]

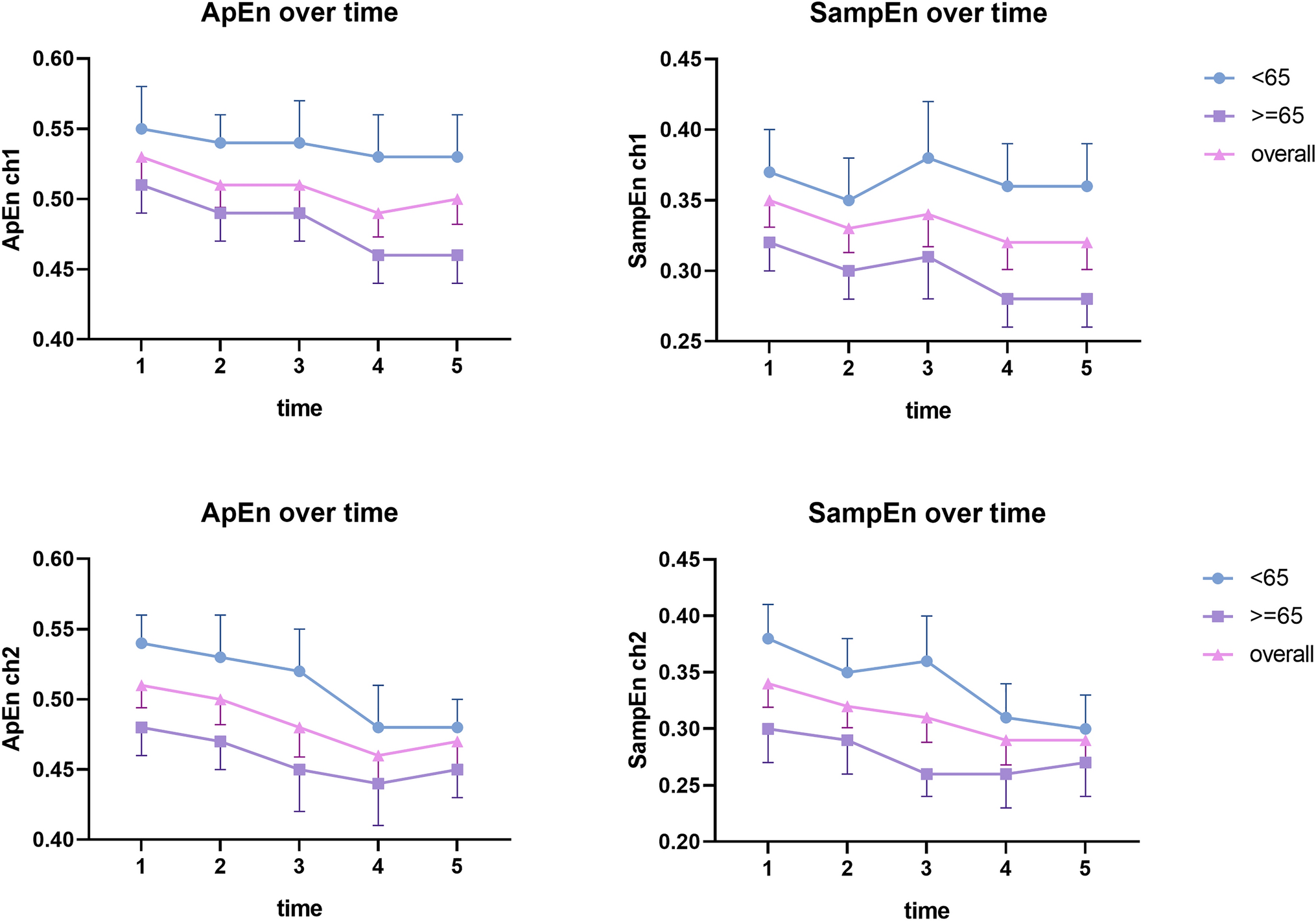

Supplement: Supplementary file 4 — Supplementary file4(PNG 375 KB) [file 40520_2022_2283_MOESM4_ESM.png]

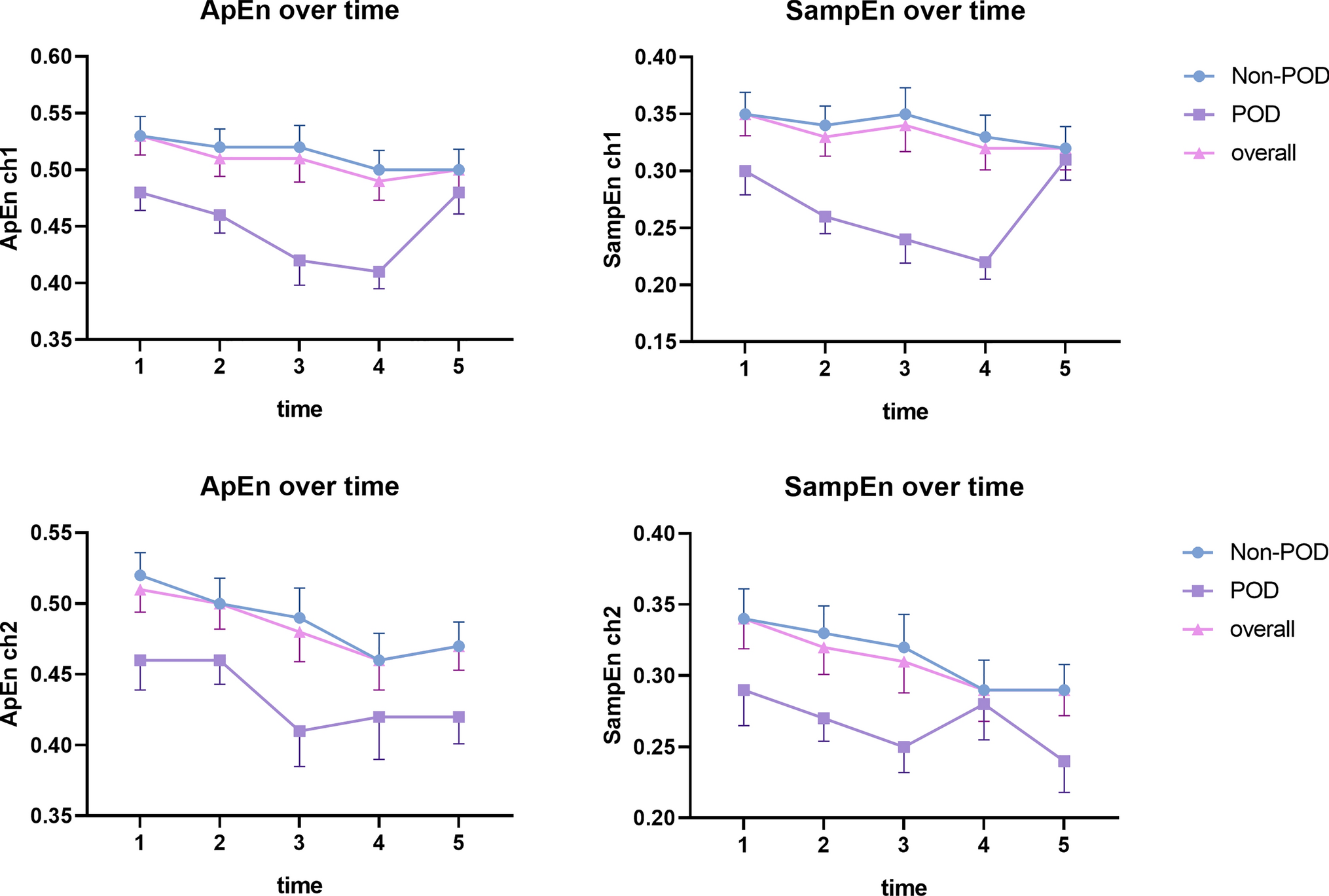

Supplement: Supplementary file 5 — Supplementary file5 (PNG 372 KB) [file 40520_2022_2283_MOESM5_ESM.png]
